# Identifying the risk factors of ICU-acquired fungal infections: clinical evidence from using machine learning

**DOI:** 10.3389/fmed.2024.1386161

**Published:** 2024-05-09

**Authors:** Yi-si Zhao, Qing-pei Lai, Hong Tang, Ren-jie Luo, Zhi-wei He, Wei Huang, Liu-yang Wang, Zheng-tao Zhang, Shi-hui Lin, Wen-jian Qin, Fang Xu

**Affiliations:** ^1^Department of Critical Care Medicine, The First Affiliated Hospital of Chongqing Medical University, Chongqing, China; ^2^Medical Data Science Academy, Chongqing Medical University, Chongqing, China; ^3^Shenzhen College of Advanced Technology, University of Chinese Academy of Sciences, Shenzhen, China; ^4^Shenzhen Institute of Advanced Technology, Chinese Academy of Sciences, Shenzhen, China

**Keywords:** fungal infection, ICU-acquired fungi, machine learning, empiric antifungal therapy, risk factors

## Abstract

**Background:**

Fungal infections are associated with high morbidity and mortality in the intensive care unit (ICU), but their diagnosis is difficult. In this study, machine learning was applied to design and define the predictive model of ICU-acquired fungi (ICU-AF) in the early stage of fungal infections using Random Forest.

**Objectives:**

This study aimed to provide evidence for the early warning and management of fungal infections.

**Methods:**

We analyzed the data of patients with culture-positive fungi during their admission to seven ICUs of the First Affiliated Hospital of Chongqing Medical University from January 1, 2015, to December 31, 2019. Patients whose first culture was positive for fungi longer than 48 h after ICU admission were included in the ICU-AF cohort. A predictive model of ICU-AF was obtained using the Least Absolute Shrinkage and Selection Operator and machine learning, and the relationship between the features within the model and the disease severity and mortality of patients was analyzed. Finally, the relationships between the ICU-AF model, antifungal therapy and empirical antifungal therapy were analyzed.

**Results:**

A total of 1,434 cases were included finally. We used lasso dimensionality reduction for all features and selected six features with importance ≥0.05 in the optimal model, namely, times of arterial catheter, enteral nutrition, corticosteroids, broadspectrum antibiotics, urinary catheter, and invasive mechanical ventilation. The area under the curve of the model for predicting ICU-AF was 0.981 in the test set, with a sensitivity of 0.960 and specificity of 0.990. The times of arterial catheter (*p* = 0.011, OR = 1.057, 95% CI = 1.053–1.104) and invasive mechanical ventilation (*p* = 0.007, OR = 1.056, 95%CI = 1.015–1.098) were independent risk factors for antifungal therapy in ICU-AF. The times of arterial catheter (*p* = 0.004, OR = 1.098, 95%CI = 0.855–0.970) were an independent risk factor for empirical antifungal therapy.

**Conclusion:**

The most important risk factors for ICU-AF are the six time-related features of clinical parameters (arterial catheter, enteral nutrition, corticosteroids, broadspectrum antibiotics, urinary catheter, and invasive mechanical ventilation), which provide early warning for the occurrence of fungal infection. Furthermore, this model can help ICU physicians to assess whether empiric antifungal therapy should be administered to ICU patients who are susceptible to fungal infections.

## 1 Introduction

Infections are been a key medical issue in in intensive care units (ICUs). In a recent survey of a worldwide sample of ICU patients, the prevalence of suspected or proven infection was 8,135 out of 15,202 (54%), and the in-hospital mortality rate was 2,404 out of 7,936 (30%) (1). Fungi are opportunistic pathogens that normally colonize the skin and mucous membranes of ICU patients (2). The entry of fungi into the body results in fungal infection when the body’s defense barrier or immune system is disrupted (3, 4). Although this decade the prevalence of fungal infection has decreased from 963 out of 4,947 (19%) to 864 out of 5,259 (16%) in the ICU, it is still the third most common pathogen in ICU (1, 5). A study reported that invasive fungal infections have a mortality rate of more than 30% in critically ill patients (6). The mortality rate after *Candida* infection is more than 40% (7, 8). Furthermore, the mortality rate attributable to invasive aspergillosis >42% (9). Fungal infections occur at different sites with varying rates. The mortality rate of patients with candidemia was 28%, which was higher than that of patients with abdominal invasive candidiasis (16%) and non-abdominal sterile sites (10%) (10). Therefore, it is important to focus on the early characteristics of fungal infections to reduce the infection and mortality rates in the ICU.

A multicenter study involving global ICU infections found that 1706 out of 8,135 (16%) infections were ICU-acquired (1), which are summed up in hospital-acquired infections (HAPs) (11). The mortality rate of ICU-acquired infections (461 out of 1706, 27%) was higher than that of community-acquired infections (697 out of 3,474, 20%) and hospital-acquired infections (661 out of 2,724, 24.9%) (1). Among the 848 (30%) cases of fungal infections, 255 were “ICU-acquired fungal infections (ICU-AFIs),” which are attributed to the special pathophysiology of critically ill patients during ICU stay (1, 8). Mainstream diagnostic methods are classified as proven, probable, and possible (12). However, diagnosing fungal infection is difficult. The false-negative rate of ICU-acquired candidemia, which is a conventional fungal infection in the ICU, can reach 60% (13). It is puzzling that the basis for the initial diagnosis of ICU-AFI limits early identification because the fungal samples belong to the ICU (255 out of 848, 30%) or other medical units (300 out of 848, 35%) (1). It is very difficult for clinical doctors to accurately confirm and treat ICU-AFI. Therefore, distinguishing between ICU-acquired fungi (ICU-AF) and non-ICU-acquired fungi (non-ICU-AF) is beneficial for the early management of ICU-AFI.

In the real world, studies on the same target may yield different results owing to multiple confounding factors. A recent study by Poissy and Keighley on the risk factors of candidemia in the ICU produced conflicting conclusions regarding urinary catheters and liver disease (14, 15). This study is a retrospective clinical cohort study that used machine learning (ML) to identify the origin of ICU-AFIs and created a scoring chart to predict ICU-AF risk models.

## 2 Methods

### 2.1 Study design

This study was approved by the Institutional Ethics Committee of the First Affiliated Hospital of Chongqing Medical University (reference number: 2021–366). The ethics committee waived the requirement for informed consent because of the retrospective nature of this study. Patient data were sourced from medical record systems and analyzed anonymously to protect patient privacy.

We included a cohort of patients who had culture-positive fungi during their admission to seven ICUs (GICU, general ICU; SICU, surgical ICU; RICU, respiratory ICU; NICU, neurology ICU; NSICU, neurosurgery ICU; CSICU, cardiothoracic surgery ICU; CCU, cardiovascular ICU) at the First Affiliated Hospital of Chongqing Medical University from January 1, 2015, to December 31, 2019. Culture-positive fungi refer to specimens obtained from ICU patients that were cultured positive for fungi by laboratory physicians in the microbiology room, and an official report was issued. Subsequently, all patient data, including basic information (age, gender and comorbidities), characteristics of fungi (microbiology and time to positivity of ICU), laboratory results (all results shall be obtained within 24 h after the fungal culture is positive), and clinical data (days in the ICU, department, Acute Physiology and Chronic Health Evaluation (APACHE) II Score, diagnosis on ICU admission, and clinical characteristics), were extracted from our internal electronic medical records (Table 1). The 28-day mortality rates after ICU admission were recorded. Data were collected by three investigators and were checked by two other investigators to avoid bias. Notably, these features were chosen on the basis of their availability in all patients rather than on any *a priori* assumptions about their ability to predict fungal acquisition, although the goal of our prediction model was to select the most influential factors in the collected data for the prediction of ICU-AF.

**Table 1:** The characteristics of training set and test set.

	Train *N* = 717	Test *N* = 717
Age	67.89 ± 15.645	68.32 ± 15.91
Gender (male)	479, 66.8%	452, 63.0%
Days in ICU	11 (5,18)	10 (5, 18)
APACHE II Score on ICU admission	20.90 ± 6.654	20.87 ± 6.05
Department		
GICU	230, 32.1%	216, 30.1%
SICU	148, 20.6%	150, 20.9%
RICU	202, 28.2%	209, 29.1%
NICU	92, 12.8%	99, 13.8%
NSICU	14, 2.0%	16, 2.2%
CSICU	13, 1.8%	8, 1.1%
CCU	18, 2.5%	19, 2.6%
Diagnosis on ICU admission		
Medical	450, 62.8%	449, 62.6%
Surgical	219, 30.5%	223, 31.1%
Trauma	48, 6.7%	45, 6.3%
Surgery	216, 30.1%	211, 29.4%
Comorbidities		
Diabetes	208, 29.0%	240, 33.5%
COPD	157, 21.9%	164, 22.9%
Heart failure	229, 31.9%	261, 36.4%
Chronic liver disease	37, 5.2%	57, 7.9%
Chronic kidney disease	68, 9.5%	70, 9.8%
Solid tumours	95, 13.2%	97, 13.5%
Haematological malignancy	5, 0.7%	9, 1.3%
Solid organ transplant	2, 0.3%	2, 0.3%
Acute pancreatitis	44, 6.1%	43, 6.0%
Sepsis	241, 33.6%	256, 35.7%
SOFA score	5.29 ± 3.06	5.38 ± 3.09
Microbiology		
Undefined Saccharomyces	185, 25.8%	191, 26.6%
*Candida albicans*	257, 33.1%	254, 35.4%
*Candida tropicalis*	75, 10.5%	86, 12.0%
Candida glabrata	88, 12.3%	83, 11.6%
*Candida parapsilosis*	24, 3.3%	24, 3.3%
*Candida krusei*	4, 0.6%	9, 1.3%
Other Candida	33, 4.6%	13, 1.8%
Undefined Aspergillus	20, 2.8%	10, 1.4%
*Aspergillus fumigatus*	30, 4.2%	30, 4.2%
Aspergillus niger	5, 0.7%	4, 0.6%
Aspergillus flavus	6, 0.8%	6, 0.8%
Other Aspergillus	1, 0.1%	1, 0.1%
*Cryptococcus neoformans*	2, 0.3%	3, 0.4%
Other fungi	6, 0.8%	3, 0.4%
Time to positivity of ICU admission over 48 h	321, 44.8%	303, 42.3%
Clinical characteristic		
Central venous catheter	482, 67.2%	464, 64.4%
Arterial catheter	399, 55.6%	389, 54.3%
Invasive mechanical ventilation	404, 56.3%	398, 55.5%
Non-invasive mechanical ventilation	318, 44.4%	323, 45.0%
Tracheotomy	127, 17.7%	124, 17.3%
Urinary catheter	617, 86.1%	604, 84.2%
Hemodialysis or continuous hemofiltration	88, 12.3%	94, 13.1%
Parenteral nutrition	433, 60.4%	414, 57.7%
Enteral nutrition	371, 51.7%	347, 48.4%
Corticosteroids	183, 25.5%	166, 23.2%
Broad-spectrum antibiotics	636, 88.7%	642, 89.5%
Candida score	1.80 ± 1.48	1.80 ± 1.47
Laboratory data		
*T*	37.05 ± 0.80	37.02 ± 0.77
*P*	100.74 ± 21.35	100.31 ± 20.49
*R*	22.79 ± 5.75	22.87 ± 5.37
WBC	12.59 ± 7.72	12.19 ± 6.55
N%	85.29 ± 8.77	84.71 ± 9.55
PLT	204.23 ± 126.61	197.28 ± 123.56
PCT	0.93 (0.23, 6.27)	1.06 (0.25, 5.46)
ALB	29.51 ± 6.31	29.81 ± 5.58
TBil	14.10 (9.50, 22.15)	14.3 (9.70, 23.55)
ALT	32.00 (22.00, 56.50)	32.00(22.00, 55.50)
AST	34.00 (22.00, 61.00)	34.00 (22.00, 59.50)
Ur	12.28 ± 9.22	12.57 ± 9.73
Cr	119.58 ± 117.72	123.31 ± 125.98
UA	272.70 ± 167.113	270.35 ± 164.375
PT	14.20 (12.80, 15.70)	14.40 (12.70, 15.65)
APTT	38.86 ± 24.76	39.38 ± 24.64
Death of 28 days	165, 23.0%	187, 26.1%

The distribution of features in the training set and test set is randomly divided equally. ICU, Intensive care unit; APACHE II, Acute Physiology and Chronic Health Evaluation II; GICU, general ICU; SICU, Surgical ICU; RICU, Respiratory ICU; NICU, Neurology ICU; NSICU, Neurosurgery ICU; CSICU, Cardiothoracic Surgery ICU; CCU, Cardiovascular ICU; COPD, Chronic obstructive pulmonary disease; SOFA, Sequential Organ Failure Assessment; T, Temperature; P, Pulse rate; R, Respiratory rate; WBC, White blood cell count; N%, Neutrophil count%; PLT, Platelet count; PCT, Procalcitonin; ALB, Albumin; TBil, Total bilirubin; ALT, Alanine aminotransferase; AST, Aspartate aminotransferase; Ur, Urea Nitrogen; Cr, Creatinine; UA, Uric acid; PT, Prothrombin time; APTT, Activated partial thromboplastin time.

### 2.2 Definition

**Figure 1:**
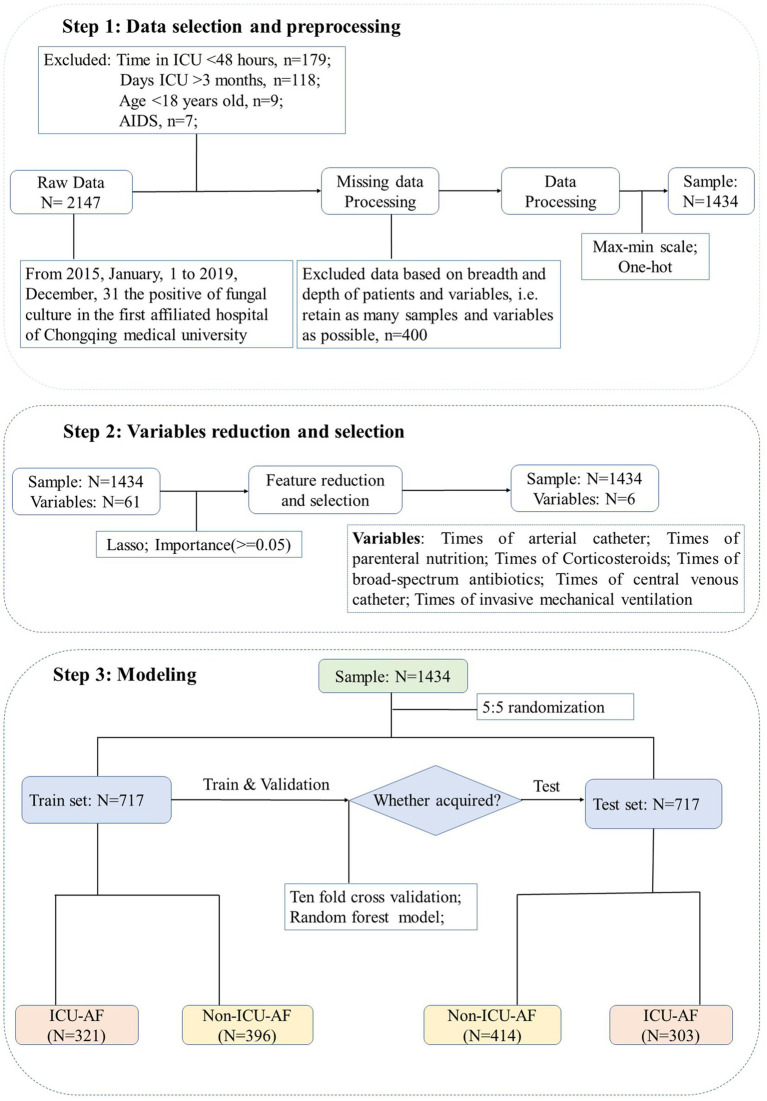
Flowchart for enrollment and screening. Step 1: Preliminarily screen the samples according to the inclusion and exclusion criteria. Step 2: Use lasso dimensionality reduction for all 61 features and select 6 features with importance ≥0.05. Step 3: All samples are randomly and equally divided into training set and test set. Max-min scale: normalization for continuous features, the formula is $x^{\prime }=\frac{x-x_{\text{min}}}{x_{\text{max}}-x_{\text{min}}}$ . One-hot: setting unordered classification features to mutually exclusive dummy features.

According to guidelines, infection after 48 h of hospitalization is defined as HAP (11). Therefore, we included patients whose first culture was positive for fungi longer than 48 h after ICU admission in the ICU-AF cohort and less than 48 h in the non-ICU-AF cohort. The cohort process and exclusion criteria are shown in Figure 1.

All antifungal treatment decisions were jointly made by two or more deputy chief physicians with >15 years of clinical experience in critical care medicine. Among these, empirical antifungal therapy prior to fungal culture is based on guidelines (6).

### 2.3 Machine learning

ML methods are computer algorithms that automatically recognize complex patterns on the basis of empirical data. The goal is to enable algorithms to learn from past or present data and to use this knowledge to make predictions or decisions regarding unknown future events (16). In the current study, we used the random forest (RF) ML algorithm. It is a “tree-based” algorithm in which multiple decision trees are constructed using random classifications of independent features that are used to predict outcome labels for random subsets of samples (17). On the one hand, the RF technique is a regression tree technique that uses bootstrap aggregation and randomization of predictor variables to achieve a high degree of predictive accuracy and is often used in medical field analysis to construct classification prediction models (18–20). On the other hand, RF may be more suitable for feature selection during classification tasks in bioinformatics and related sciences, where it has a relatively low tendency to overfit and produces more robust results (21).

### 2.4 Data set division

We randomly assigned 1,434 cases to the sample, with 50% of the cases used as the training set and the rest as the test set. We also ensured that there was no gender or age bias between the training set and testing set.

### 2.5 Feature extraction

For the training set, we first used the Least Absolute Shrinkage and Selection Operator (LASSO) to reduce the dimension of features according to whether the patient is ICU-AF. We performed feature reduction using LASSO on the training set. LASSO performs feature selection during model construction by penalizing the respective regression coefficients. As this penalty increases, more regression coefficients shrink to zero, thus resulting in a more regularized model (22). In this process, 49 significant features with nonzero coefficients were obtained. We then used them in the RF prediction model. By using a ten-fold cross-validation analysis, we selected the best model parameter on the basis of the accuracy of each fold of the model. At the same time, we ranked the features in this model by setting a threshold of 0.05 to select the features in reference to previous articles (23, 24). These features were retained, and the randomized forest model was trained to predict patient ICU-AF by using ten-fold cross-validation. Finally, the model was tested using the test set. Both downscaling and ten-fold cross-validation were used to prevent overfitting. Overfitting can occur when excessive features affect the predictive performance of a model. However, the use of nested k-fold cross-validation allows us to perform model training independently of hyperparameters optimization, which prevents overfitting or incorrect generalization estimates (25). The R language was used to draw the density map between each feature and APACHE II, and the *lm* function was used to fit the regression model. The “pheatmap” package implements heatmap to display sample survival and feature performance.

### 2.6 Model performance

In the ten-fold cross-validation of the model, we trained different model parameters and selected the model parameters with the best accuracy in one fold for application to the test set. The ability of the model to discriminate between acquired fungi was determined using the area under the curve (AUC), and the stability of the model was determined on the basis of sensitivity and specificity. From our learning models, we chose the model with the best discrimination ability.

### 2.7 Statistical analysis

All statistical analyses were performed using Stata 24.0 software. To divide the training and test sets, we used analysis of variance (ANOVA) to analyze whether there was a difference in the age distribution between the training and test sets, and the chi-square test was used to analyze whether there was a difference in the gender distribution between them. The main specification of ML is that the models constructed from selected features perform well for predicting patient outcomes, AUC, sensitivity, specificity, and accuracy and are only used to determine the performance of the models (26). Therefore, many previous studies have used ML and logic methods (27, 28). In our research, factors associated with antifungal therapy and empirical antifungal therapy for acquired fungi were analyzed using univariate and multivariate conditional logistic regression models for all features of the ML model. Its odds ratio (OR) and 95% confidence interval (CI), *p* < 0.05 was considered significant.

## 3 Results

### 3.1 Cohort characteristics

**Figure 2:**
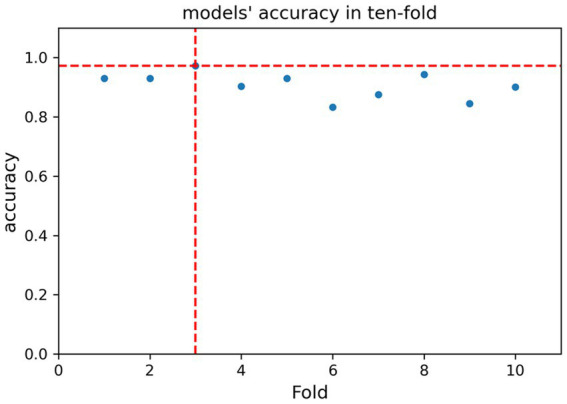
Accuracy of models in ten-fold cross-validation.

**Figure 3:**
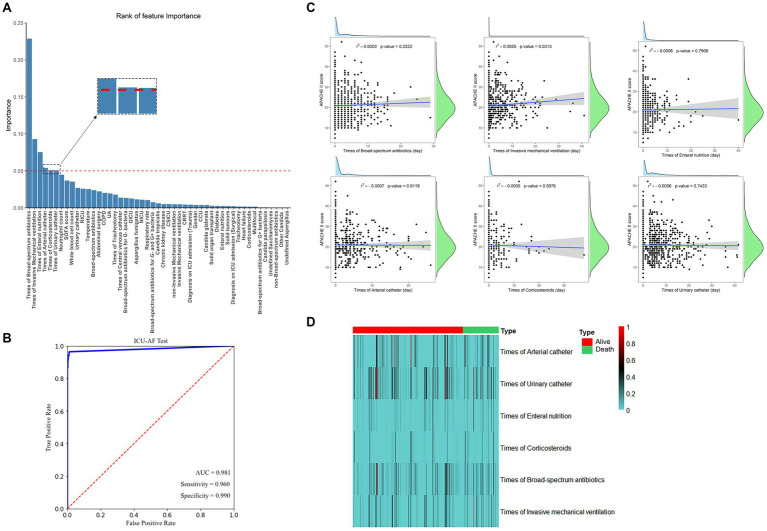
**(A)** The 49 features with the highest relative gain for model predicting ICU-AF and the 5 features with importance ≥0.05. **(B)** Receiver operating characteristic curve (ROC) of models. **(C)** Scatter plot with linear regression line of best fit with APACHE II score analyzed separately with six features. *r*^2^: represents the degree of feature fitting; *p* < 0.05 were considered significant; **(D)** The heatmap of different features in dead vs. surviving patients, and colors in the heatmap indicate the time (days) for the corresponding feature. AUC, area under the subject curve. APACHE II, Acute Physiology and Chronic Health Evaluation II.

For the submission of the manuscript, we enrolled 2,147 cases. A total of 1,434 cases with complete data were obtained after exclusion and screening (Figure 1, step 1). The cases were randomly and equally divided into the training (*N* = 717) and test (*n* = 717) sets. The distribution of outcome labels for patients in the training and test sets showed no significant differences (*p* = 0.37, chi-square test, not shown). The features of the two data sets are shown in Table 1; age (ANOVA, *p* = 0.60, not shown) and gender (chi-square test, *p* = 0.15, not shown) had no statistical difference, and the other features are shown in Table 1. On the basis of whether the fungi were ICU acquired, LASSO was performed to reduce the dimension of features. Thereafter, by using ten-fold cross-validation, the average accuracy of the random forest model was 0.907 ± 0.042, among which the third-fold accuracy we applied was the highest, which was 0.972 (Figure 2). We took the third-fold model parameter as our optimal model parameter. We selected six features with importance ≥0.05 in the optimal mode, namely, times of arterial catheter, times of enteral nutrition, times of corticosteroids, times of broad-spectrum antibiotics, times of urinary catheter and times of invasive mechanical ventilation (Figure 3A).

### 3.2 The role of each feature

By using these features for ML analysis and testing on an independent test set, the results showed that the AUC for predicting ICU-AF was 0.981 in the test set, with a sensitivity of 0.960 and specificity of 0.990 (Figure 3B). Disease severity in ICU patients was represented by the APACHE II Score, which was analyzed separately with the continuous time of these six features. Only the times of invasive mechanical ventilation showed a significant linear correlation with the APACHE II Score (*p* = 0.031) (Figure 3C). The duration time of these features showed no significant differences in the 28-day mortality (Figure 3D).

### 3.3 Risk factors associated with antifungal therapy and empirical antifungal therapy

Considering the univariate and multivariate conditional logistic regression analyses of antifungal therapy in ICU-AF, the results showed that among these six features, times of arterial catheter (*p* = 0.011, OR = 1.057, 95%CI = 1.053–1.104) and times of invasive mechanical ventilation (*p* = 0.007, OR = 1.056, 95%CI = 1.015–1.098) were independent risk factors for antifungal therapy in ICU-AF (Table 2). In the sample on antifungal therapy, times of arterial catheter (*p* = 0.004, OR = 1.098, 95%CI = 0.855–0.970) was an independent risk factor for empirical antifungal therapy (Table 3).

**Table 2:** Independent risk factors associated with antifungal therapy according to ICU-AF.

Variable (Times of)	Univariate			Multivariate		
	OR	95%CI	*p* value	OR	95%CI	*p* value
Arterial catheter	1.061	1.021–1.101	0.002	1.057	1.013–1.104	0.011
Invasive mechanical ventilation	1.056	1.023–1.089	0.001	1.056	1.015–1.098	0.007
Urinary catheter	1.028	1.003–1.053	0.027	0.984	0.950–1.020	0.378
Parenteral nutrition	1.036	0.989–1.085	0.136	1.01	0.957–1.066	0.718
Corticosteroids	1.029	0.980–1.080	0.253	0.999	0.947–1.054	0.978
Broad-spectrum antibiotics	1.037	0.996–1.079	0.077	1.005	0.959–1.052	0.846

OR stands for odds ratio, CI for confidence interval; The samples for logistic regression analysis were from all ICU-AF samples. Variables were selected with importance ≥ 0.05 in the training set.

**Table 3:** Independent risk factors associated with empirical antifungal therapy according to ICU-AF.

Variable (Times of)	Univariate			Multivariate		
	OR	95%CI	*p* value	OR	95%CI	*p* value
Arterial catheter	1.088	1.035–1.143	0.001	1.098	0.855–0.970	0.004
Invasive mechanical ventilation	1.028	0.987–1.070	0.188	1.053	0.894–1.009	0.094
Urinary catheter	1.018	0.980–1.057	0.362	0.947	0.992–1.125	0.089
Parenteral nutrition	0.99	0.924–1.060	0.769	0.98	0.931–1.118	0.666
Corticosteroids	1.039	0.971–1.111	0.265	1.015	0.910–1.066	0.714
Broad-spectrum antibiotics	1.068	1.007–1.134	0.028	1.052	0.884–1.023	0.176

OR stands for odds ratio, CI for confidence interval; The samples for logistic regression analysis were from all ICU-AF samples with antifungal therapy. Variables were selected with importance ≥ 0.05 in the training set.

## 4 Discussion

This retrospective clinical cohort study spanned 5 years, included 1,434 cases with complete data, and identified 6 risk factors for ICU-AF using ML. Fungal infection, which is accompanied by difficult treatment and poor prognosis, is an important component of ICU infections (1, 29). He et al. used ML to establish predictive models for secondary candidemia in patients with systemic inflammatory response syndrome (SIRS) patients in the ICU. These models have a potential guiding role in the antifungal treatment of critically ill patients with SIRS (30). Researchers often focus on the pathogenic state and non-pathogenic state of fungi, which are known as “infection” and “colonization,” respectively (31, 32). Once a fungal infection emerges in critically ill patients in the ICU, colonization poses a high risk to individuals with immune disorders. Popular researches has considered fungal colonization, including multi-site colonization and the colonization of special strains, as a risk factors for fungal infection (33, 34). The risk of fungal infection increased significantly after fungal colonization in ICU patients. One study found that 93 out of 137 (68%) patients with candidemia had *Candida* colonization (30). The preconception was that fungal infection is opportunistic. However, the sensitivity of ICU blood cultures for invasive candidiasis (including intra-abdominal candidiasis) is approximately 40% (13). Up to 70% of patients with candidemia do not receive early empiric antifungal therapy early on (35). Generally, doctors in the ICU often value patients who already have the “fungi” label, but the preparation for a new onset one is insufficient. This could increase the risk of patients in the ICU. A study showed that a 12-h delay in starting antifungal therapy was associated with a 2.09-fold increase in mortality (36). Discovering the types of patients in the ICU who are at high risk for acquired fungal infections is an important part of critical illness warnings.

This study advances the warning line of fungal infection before colonization, which is called ICU-AF and is defined as fungi cultured after 48 h in the ICU. LASSO dimensionality reduction and ML methods were used to analyze patients admitted to the ICU over the past 5 years. Compared with non-acquired fungi, six features including times of arterial catheter, times of enteral nutrition, times of corticosteroids, times of broad-spectrum antibiotics, times of urinary catheter, and times of invasive mechanical ventilation, showed high significance in ICU-AF. These features are considered high-risk factors for fungal infection in the ICU (7, 37–42). The current study used ML to prove that ICU-AF has a higher risk of occurrence when ICU patients exhibit the above six features. However, the utility of risk factors in ICU-AF patients depends on differentiating between the dimensions of time, frequency, and intensity. ICU-AF is expected to provide an early warning for antifungal therapy or even empirical antifungal therapy.

Logistic regression analysis showed that the times of arterial catheter and invasive mechanical ventilation were independent risk factors for antifungal therapy in ICU-AF, and ductus arteriosus time was an independent risk factor for empirical antifungal therapy in ICU-AF. By using ML to study the early warning of ICU-AF, the times of arterial catheter insertion and invasive mechanical ventilation can be used to warn critical care physicians on whether antifungal therapy is needed. Patients with arterial catheters may require early empirical antifungal therapy.

### 4.1 Strengths and limitations

This study applied an unconventional method to study susceptibility to ICU-AF: First, we used efficient ML methods to analyze clinical data to reduce the bias of manual analysis. Second, we focused on the early warning of fungal infection, namely, ICU-AF, and this approach is more in line with the needs of treating ICU patients. Finally, we investigated the role of the ICU-AF early warning model in antifungal therapy and empirical antifungal therapy for guiding the management of ICU-AF.

This study has the following limitations. First, this was a retrospective, single-center study. It should be noted that this single-center study involved seven different ICU wards (GICU, RICU, SICU, NICU, NSICU, CSICU, and CCU), and some specific characteristics (such as major abdominal surgery and disturbance of consciousness) were diverse. However, even across seven different ICUs, each patient had these six features. As a routine treatment procedure for patients in the ICU, the duration of these six features was obtained via detailed nursing records and reflected the length of time that patients received treatment in the real world and the homogeneity of fungal infection risk factors across all ICUs. Second, there were more than 40 salient features in the optimal model (Figure 3A). However, we selected only six features with importance >0.05. When multiple features appear in the results, it is crucial to extract better and more convenient feature models for clinical applications. In addition to using 0.05 as a threshold to screen six features, we also explored the important role of these six features in ICU-AF on the basis of clinical practice. Other features (such as central venous catheter, abdominal surgery and SOFA scores) that were reported to be connected with ICU-AF (43, 44), could probably have hidden roles. They still have potential value for future discussion. The prevailing view supports that the six features, analyzed in the current study are good predictors of ICU-AF (7, 37–42). Controlling these six operations is an effective way to reduce ICU-AF. Blaize et al. found that controlling the use of corticosteroids could reduce the risk of invasive pulmonary fungal infections in COVID-19 patients admitted to the ICU (45). Thirdly, regarding the question of whether ICU physicians can distinguish fungal colonization from fungal infection. The AUC of the optimal model for the fungal infection test obtained in this study was 0.670 (Supplementary Figure S1). In the clinical cohort, these were indistinguishable at the time of diagnosis; thus, we advanced the field of view to the acquired fungus. Finally, increasing the amount of training data can enable us to obtain more information and make diverse learning in most cases, as well as increase the chances of achieving better results. Some important studies use 70% or 80% of samples in the training set (46–48). We randomly assigned 1,434 cases to the sample, with 50% of the cases used as the training set and the rest as the test set, to improve the efficiency of model validation. Meanwhile, it was also ensured that there was no gender and age bias between the training set and the test set. Although this ratio is also a common ratio for dividing datasets in previous studies, such as in some studies on tumor diseases (49, 50), we will continue to collect and expand sample size data in future research to improve the sample ratio in the training set.

In summary, ML classifier models in clinical cohorts have the potential to predict the risk of ICU-AFI. The most important risk factors for ICU-AF are the six time-related clinical parameters (arterial catheter, enteral nutrition, corticosteroids, broad-spectrum antibiotics, urinary catheter, and invasive mechanical ventilation) that provide early warnings for the early prevention of fungal infection. Furthermore, this model, although needs to be more clinically validated, has the potential to help ICU physicians assess whether empiric antifungal therapy should be administered to ICU patients who are susceptible to fungal infections.

## Data availability statement

The raw data supporting the conclusions of this article will be made available by the authors, without undue reservation.

## Ethics statement

The studies involving humans were approved by Institutional Ethics Committee of the First Affiliated Hospital of Chongqing Medical University. The studies were conducted in accordance with the local legislation and institutional requirements. Written informed consent for participation was not required from the participants or the participants' legal guardians/next of kin in accordance with the national legislation and institutional requirements.

## Author contributions

Y-sZ: Data curation, Formal analysis, Funding acquisition, Investigation, Methodology, Project administration, Writing – original draft. Q-pL: Formal analysis, Methodology, Software, Supervision, Writing – original draft. HT: Conceptualization, Data curation, Investigation, Resources, Writing – original draft. R-jL: Data curation, Methodology, Writing – original draft. Z-wH: Data curation, Investigation, Software, Writing – original draft. WH: Data curation, Investigation, Writing – original draft. l-yW: Data curation, Writing – original draft. Z-tZ: Investigation, Writing – original draft. S-hL: Data curation, Investigation, Resources, Writing – original draft. W-jQ: Conceptualization, Methodology, Project administration, Resources, Software, Supervision, Writing – review & editing. FX: Conceptualization, Funding acquisition, Project administration, Resources, Supervision, Validation, Visualization, Writing – review & editing.
