# Supplementary material for: Identifying the risk factors of ICU-acquired fungal infections: clinical evidence from using machine learning
Source: Front Med (Lausanne). 2024 May 9;11:1386161. doi: 10.3389/fmed.2024.1386161 (PMC11112035; doi:10.3389/fmed.2024.1386161)
Supplement: Supplementary file 1 [file Data_Sheet_1.docx]

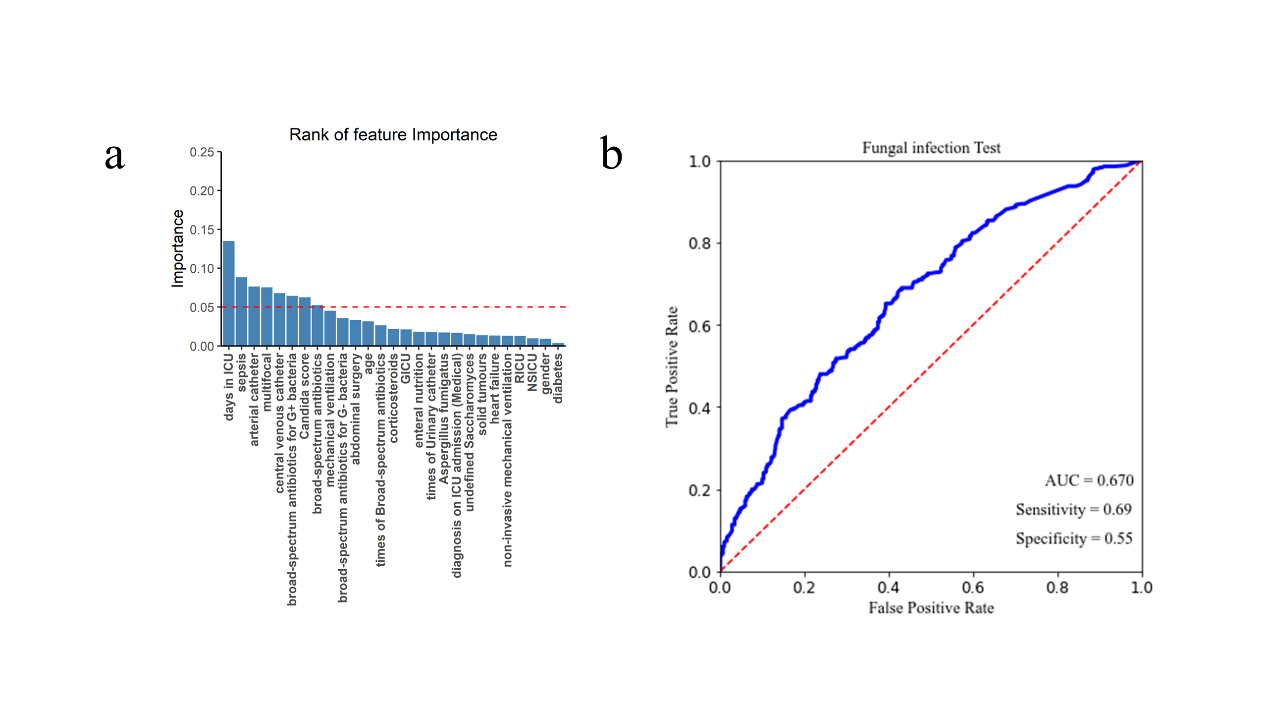


Supplementary Figure 1. The Model of Fungal Infection

(a) The features with the highest relative gain for model predicting fungal infection and the 8 features with importance ≥ 0.05. (b). Receiver operating characteristic curve (ROC) of fungal infection models.
